# Evaluation of perioperative D-dimer concentration for predicting postoperative deep vein thrombosis following hepatobiliary-pancreatic surgery

**DOI:** 10.1007/s00595-023-02645-5

**Published:** 2023-01-29

**Authors:** Teruhisa Sakamoto, Yuki Murakami, Takehiko Hanaki, Kyoichi Kihara, Tomoyuki Matsunaga, Manabu Yamamoto, Shuichi Takano, Naruo Tokuyasu, Toshimichi Hasegawa, Yoshiyuki Fujiwara

**Affiliations:** Division of Gastrointestinal and Pediatric Surgery, Department of Surgery, School of Medicine, Faculty of Medicine, Tottori University, 36-1 Nishi-cho, Yonago, 683-8504 Japan

**Keywords:** D-dimer, Deep vein thrombosis, Hepatectomy

## Abstract

**Purpose:**

This study was performed to investigate the predictive value of the perioperative D-dimer concentration for the development of postoperative deep vein thrombosis (DVT) after hepatobiliary-pancreatic (HBP) surgery.

**Methods:**

The subjects of this retrospective study were 178 patients who underwent HBP surgery in our hospital between January, 2017 and December, 2021. The D-dimer concentration was measured preoperatively and on postoperative days (POD) 1, 3, and 5. Postoperative DVT was diagnosed based on compression ultrasonography in both lower limbs on POD 6 or 7.

**Results:**

Postoperative DVT developed in 21 (11.8%) of the 178 patients. The D-dimer concentration was significantly higher in the patients with than in those without postoperative DVT before surgery and on PODs 1, 3, and 5. The highest area under the curve of the D-dimer concentration for predicting DVT was 0.762 on POD 3. Multivariate analysis revealed that the D-dimer concentration on POD 3 was an independent predictive risk factor for postoperative DVT, along with the preoperative estimated glomerular filtration rate. Preoperative albumin and D-dimer concentrations were also identified as independent predictive factors of an increase in D-dimer concentration on POD 3.

**Conclusions:**

The D-dimer concentration on POD 3 is a useful predictor of DVT after HBP surgery.

## Introduction

Venous thromboembolism (VTE), including deep vein thrombosis (DVT) and pulmonary embolism (PE), is a postoperative complication with multifactorial causes following gastroenterological surgery. In a recent survey on VTE following major gastroenterological surgeries in Japan, the frequency of postoperative DVT and PE was 0.3% (range, 0.1–0.7%) and 0.2% (range, 0.1–0.3%), respectively, and the incidence rate of VTE was similar to that in Western countries [1]. Hepatobiliary-pancreatic (HBP) surgeries, mainly represented by major hepatectomy or pancreaticoduodenectomy, are often highly invasive procedures with a long operative duration; thus, the incidence of VTE following these procedures is higher than that following other gastroenterological surgeries, with the exception of esophagectomy [1, 2]. Although postoperative VTE occurs at a lower rate than other major complications of HBP surgeries, such as pancreatic fistula and bile leakage, it can be life-threatening. PE is potentially fatal and manifests as sudden symptoms, such as dyspnea, chest pain, hypoxemia, and low blood pressure. However, PE is frequently difficult to diagnose early and is often caused by DVT. Therefore, identification of the predictive risk factors for the development of DVT during the perioperative period is critically important to prevent a life-threatening event after HBP surgery.

D-dimer is a marker of endogenous fibrinolysis and is detectable in patients with DVT [3]. Measurement of the D-dimer concentration during the perioperative period can be useful for the diagnosis of DVT in clinical practice and is reportedly a predictive factor for the development of DVT after gastrointestinal and HBP surgery [4, 5]. However, the predictive value of D-dimer measurement during the perioperative period for the early diagnosis of DVT after HBP surgery remains unclear. Moreover, no comparative studies have been performed to identify which HBP surgical procedures are associated with a high risk of DVT.

We conducted the present study to investigate the predictive value of the D-dimer concentration during the perioperative period for the development of early postoperative DVT after HBP surgery and to clarify which HBP surgeries have a high risk of postoperative DVT.

## Methods

### Patients

We reviewed the medical records of 202 patients who underwent HBP surgery, excluding simple cholecystectomy, at our institution between January, 2017 and December, 2021. After the exclusion of 24 patients for whom data was unavailable, 178 patients were enrolled in this study. The institutional review board of Tottori University approved the study (Approval No. 21A125) and the requirement for informed consent was waived because of the retrospective nature of the study.

### Intra- and postoperative prophylaxis for DVT

The patients wore intermittent pneumatic compression devices or elastic compression stockings from the induction of general anesthesia to the morning of POD 1 for prophylaxis of postoperative DVT. Postoperative ambulation was initiated on POD 1. None of the patients received routine prophylactic anticoagulation for DVT.

### Evaluation of DVT

**Fig. 1:**
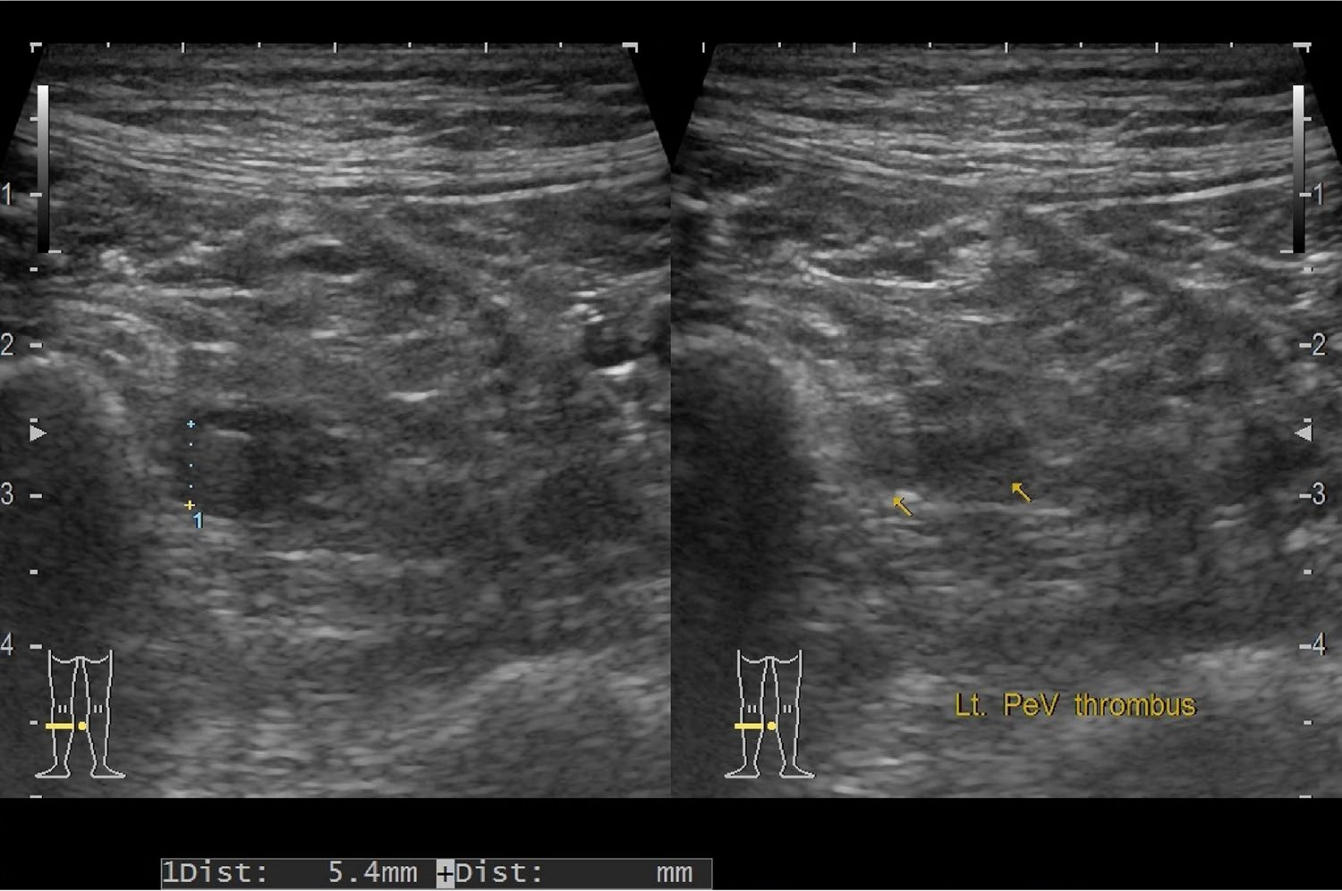
Compression ultrasonography image showing deep vein thrombosis in the peroneal vein. The number “1” indicates the size of thrombosis and the yellow arrows indicate the thrombosis in the left peroneal vein. *lt* left, *PeV* peroneal vein

The patients underwent compression ultrasonography in both lower limbs to screen for postoperative DVT by two experienced sonographers on postoperative day (POD) 6 or 7. Postoperative DVT was diagnosed according to the presence of fresh thrombosis with a constant intraluminal filling defect or lack of vein compression (Fig. 1).

### Clinical characteristics

The following clinical characteristics were collected from the patients’ records: age, sex, body mass index, surgical procedures, histological diagnosis (malignancy or not), American Society of Anesthesiologists classification, VTE risk classification, comorbidities, antithrombotic medication before surgery, preoperative albumin concentration, preoperative platelet count, preoperative C-reactive protein concentration, preoperative prothrombin time, preoperative estimated glomerular filtration rate (eGFR), operative time, and intraoperative blood loss volume. In this study, with respect to the risk classification of DVT, we defined high or highest risk as “high” and intermediate or low risk as “low” in accordance with the Japanese Circulation Society guidelines for VTE [6]. The D-dimer concentration was measured within 7 days prior to surgery and on PODs 1, 3, and 5.

### Statistical analysis

Continuous variables are expressed as means and the standard deviation. To assess between-group differences in the clinical variables, the chi-square test or Fisher’s exact probability test was used for categorical variables and the Mann–Whitney U test was used for continuous variables. Receiver operating characteristic (ROC) analysis was performed to evaluate the area under the curve (AUC) of the D-dimer concentration for the prediction of postoperative DVT and the optimal cutoff value of the D-dimer concentration was determined using Youden’s index in the ROC analysis. Univariate and multivariate logistic regression analyses were performed to clarify predictive risk factors for postoperative DVT following HBP surgery. A *P* value of < 0.05 was considered significant. All statistical analyses were performed using SPSS Version 24 (IBM Corp., Armonk, NY, USA).

## Results

Table 1 summarizes the patients’ clinical characteristics. Postoperative DVT developed in 21 (11.8%) of the 178 patients. All patients with postoperative DVT were asymptomatic. Chest enhanced computed tomography, which was performed to screen for pneumonia 1 week after surgery, revealed concomitant asymptomatic PE in one patient. We administered unfractionated heparin intravenously and then changed the anticoagulant therapy to oral edoxaban. No patients with postoperative DVT suffered serious complications during their postoperative clinical course. The incidence of DVT among all hepatobiliary surgeries was higher than that among all pancreatectomies (29.6% vs. 8.4%, respectively). Of the 21 patients with postoperative DVT, 12 (57.1%) had undergone hepatectomy. The incidence of postoperative DVT was highest after hepatopancreatoduodenectomy (2/4, 50.0%) and hepatectomy (S4a + S5 resection or hemihepatectomy + caudate lobectomy) with extrahepatic bile duct resection (4/13, 30.8%). In contrast, postoperative DVT did not develop in any of the patients who underwent extrahepatic bile duct resection with cholecystectomy, which is regarded as a minimally invasive surgery. With respect to the type of surgery, only hepatectomy with extrahepatic bile duct resection, including hepatopancreatoduodenectomy, was significantly associated with postoperative DVT (odds ratio [OR], 7.350; 95% confidence interval [CI], 2.421–22.316; *P* < 0.001, Table 2).

**Table 1:** Clinical characteristics of the patients who underwent hepatobiliary-pancreatic surgery

Characteristics	*n *= 178
Postoperative DVT, *n* (%)	21 (11.8)
Incidence of DVT by surgical procedure	
Hepatobiliary surgery, *n* (%)	21/71 (29.6)
Hepatectomy (S4a + S5 resection or hemihepatectomy + caudate lobectomy) with extrahepatic bile duct resection, *n* (%)	4/13 (30.8)
Hemihepatectomy, *n* (%)	1/8 (12.5)
Hepatic sectionectomy, *n* (%)	2/13 (15.4)
Hepatic segmentectomy, *n* (%)	2/7 (28.6)
Hepatic partial resection, *n* (%)	1/19 (5.3)
Extrahepatic bile duct resection with cholecystectomy (with or without gallbladder bed resection), *n* (%)	0/7 (0.0)
Hepatopancreatoduodenectomy, *n* (%)	2/4 (50.0)
Pancreatic surgery, *n* (%)	9/107 (8.4)
Pancreatoduodenectomy, *n* (%)	7/71 (9.9)
Distal pancreatectomy, *n* (%)	2/36 (5.6)
Age, years	69.3 ± 11.7
Male sex, *n* (%)	112 (62.9)
Body mass index prior to surgery, kg/m^2^	23.0 ± 4.1
Malignant diagnosis, *n* (%)	148 (83.1)
ASA classification, *n* (%)	
1	11 (6.2)
2	113 (63.5)
3	53 (29.8)
4	1 (0.6)
Comorbidities	
Hypertension, *n* (%)	97 (54.5)
Cardiac disease, *n* (%)	20 (11.2)
Respiratory disease, *n* (%)	5 (2.8)
Diabetes mellitus, *n* (%)	54 (29.2)
Antithrombotic medication before surgery, *n* (%)	19 (10.7)
Preoperative serum albumin, g/dL	4.0 ± 0.4
Preoperative platelet count, × 10^3^/μL	215.6 ± 74.1
Preoperative CRP, mg/dL	0.51 ± 1.25
Preoperative PT, %	97.1 ± 15.7
Preoperative eGFR, mL/min/1.73 m^2^	72.2 ± 19.7
D-dimer before surgery, μg/mL	1.66 ± 3.09
D-dimer on POD 1, μg/mL	6.40 ± 3.96
D-dimer on POD 3, μg/mL	6.80 ± 5.49
D-dimer on POD 5, μg/mL	10.89 ± 7.63
Operative time, min	458 ± 187
Intraoperative blood loss volume, mL	616 ± 568

Continuous variables are expressed as means ± standard deviation

*DVT* deep vein thrombosis, *ASA* American Society of Anesthesiologists, *CRP* C-reactive protein, *PT* prothrombin time, *eGFR* estimated glomerular filtration rate, *POD* postoperative day

**Table 2:** Risk of postoperative deep vein thrombosis according to the type of surgery

Type of surgery	Odds ratio	95% CI	*P *value
Hepatectomy with extrahepatic bile duct resection, including hepatopancreatoduodenectomy	7.350	2.421–22.316	< 0.001
Hemihepatectomy	1.071	0.125–9.167	0.950
Hepatic sectionectomy	1.397	0.288–6.787	0.678
Hepatic segmentectomy	3.200	0.580–17.653	0.182
Hepatic partial resection	2.590	0.651–4.795	0.367
Pancreatoduodenectomy	1.766	0.328–20.474	0.264
Distal pancreatectomy	2.626	0.583–11.836	0.209

*DV* deep vein thrombosis

Table 3 compares the clinical characteristics between the patients with and those without postoperative DVT. There were significant correlations between the two groups with respect to age; VTE risk classification; preoperative serum albumin concentration; preoperative eGFR; D-dimer concentration before surgery and on PODs 1, 3, and 5; operative time; and hepatectomy with extrahepatic bile duct resection, including hepatopancreatoduodenectomy. The mean age and rate of high risk by VTE risk classification for postoperative DVT were significantly higher for the patients with DVT than for those without DVT. The D-dimer concentration was significantly higher for the patients with postoperative DVT than for those without postoperative DVT at all time points: before surgery and on PODs 1, 3, and 5. The D-dimer concentration increased gradually from the preoperative period to POD 5 in the patients with and those without postoperative DVT. The D-dimer concentration was highest on POD 5 in the patients with and those without postoperative DVT. However, the disparity of the D-dimer concentration between the patients with and those without postoperative DVT on POD 3 was greatest among all the time points. The incidence of hepatectomy (S4a + S5 resection or hemihepatectomy + caudate lobectomy) with extrahepatic bile duct resection, including hepatopancreatoduodenectomy, was significantly higher in the patients with than in those without DVT. In contrast, both the preoperative albumin concentration and preoperative eGFR were significantly lower in the patients with than in those without postoperative DVT.

**Table 3:** Clinical characteristics of patients with vs. those without deep vein thrombosis

Characteristics	DVT in early postoperative period		*P* value
	Present (*n* = 21)	Absent (*n* = 157)	
Age, years	74.3 ± 5.0	68.6 ± 12.2	0.019
Male sex	12 (57.1)	100 (63.7)	0.559
Body mass index, kg/m^2^	22.3 ± 2.4	23.1 ± 4.2	0.473
Malignant diagnosis	20 (95.2)	128 (81.5)	0.209
ASA classification, 1 or 2	16 (76.2)	108 (68.8)	0.488
Risk classification of VTE, high	21 (100.0)	0 (0.0%)	0.049
Hypertension	15 (71.4)	82 (52.2)	0.097
Cardiac disease	3 (14.3)	17 (10.8)	0.711
Respiratory disease	1 (4.8)	4 (2.5)	0.470
Diabetes mellitus	5 (23.8)	47 (29.9)	0.799
Antithrombotic medication	4 (19.0)	15 (9.6)	0.248
Preoperative serum albumin, g/dL	3.8 ± 0.4	4.1 ± 0.4	0.006
Preoperative platelet count, × 10^3^/μL	245.2 ± 93.9	211.6 ± 70.5	0.151
Preoperative CRP, mg/dL	0.83 ± 1.61	0.47 ± 1.19	0.550
Preoperative PT, %	90.7 ± 18.6	98.0 ± 15.2	0.096
Preoperative eGFR, mL/min/1.73 m^2^	61.4 ± 17.2	73.6 ± 19.6	0.005
D-dimer before surgery, μg/mL	3.39 ± 4.93	1.44 ± 2.70	< 0.001
D-dimer on POD 1, μg/mL	8.90 ± 5.38	6.06 ± 3.62	0.011
D-dimer on POD 3, μg/mL	12.14 ± 9.25	6.09 ± 4.34	< 0.001
D-dimer on POD 5, μg/mL	17.61 ± 14.47	9.99 ± 5.68	0.021
Operative time, min	549 ± 232	446 ± 178	0.040
Intraoperative blood loss volume, mL	834 ± 684	587 ± 547	0.066
Hepatectomy with extrahepatic bile duct resection, including hepatopancreatoduodenectomy	7 (33.3)	10 (6.4)	< 0.001

Data are expressed as means ± standard deviation or *n* (%)

*DVT* deep vein thrombosis, *ASA* American Society of Anesthesiologists, *VTE* venous thromboembolism, *CRP* C-reactive protein, *PT* prothrombin time, *eGFR* estimated glomerular filtration rate, *POD* postoperative day

**Fig. 2:**
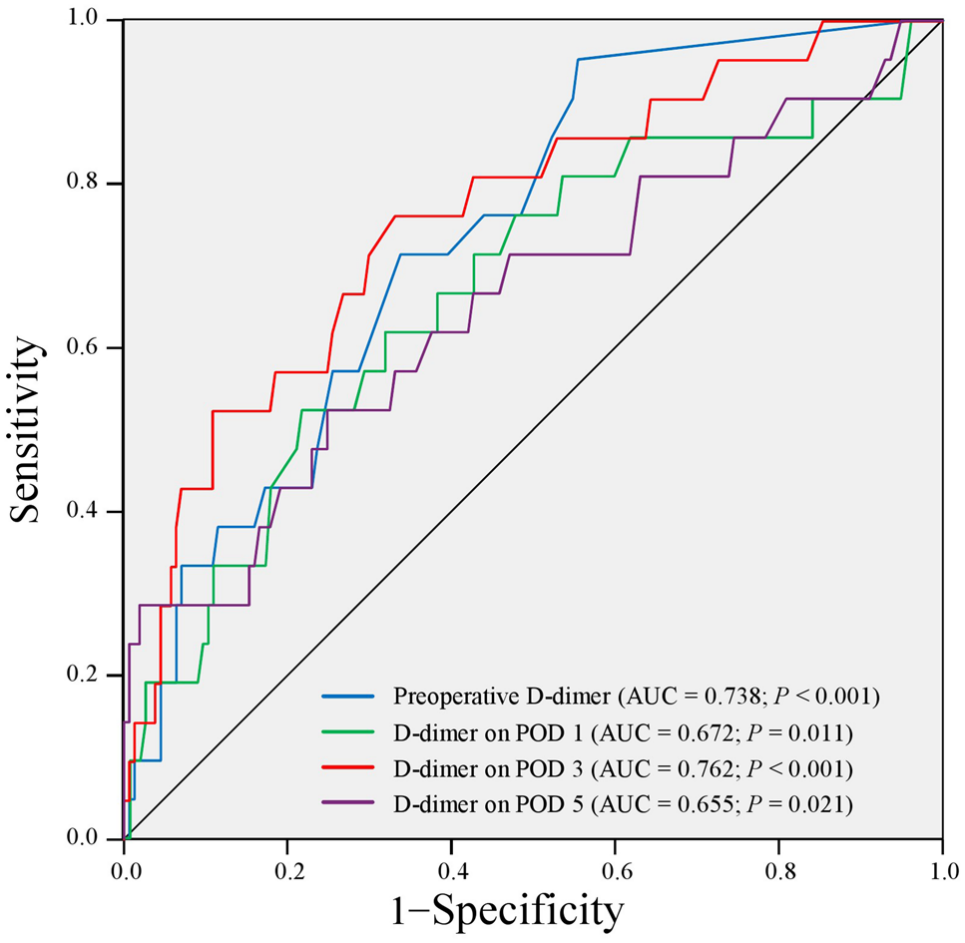
Areas under the curve of the D-dimer concentration for predicting postoperative deep vein thrombosis in patients who underwent hepatobiliary-pancreatic surgery *AUC* area under the curve; *POD* postoperative day

Based on the highest Youden indices of the ROC analysis, the optimal cutoff values of the D-dimer concentration were as follows: 0.45 before surgery, 5.35 on POD 1, 6.25 on POD 3, and 12.10 on POD 5. ROC analysis revealed that the AUC of the D-dimer concentration for the discrimination ability to predict postoperative DVT was 0.762 (*P* < 0.001) on POD 3, which was the highest value among all AUCs of the D-dimer concentrations measured in this study (0.738 before surgery, *P* < 0.001; 0.672 on POD 1,* P* = 0.001; and 0.655 on POD 5, *P* = 0.021) (Fig. 2).

**Fig. 3:**
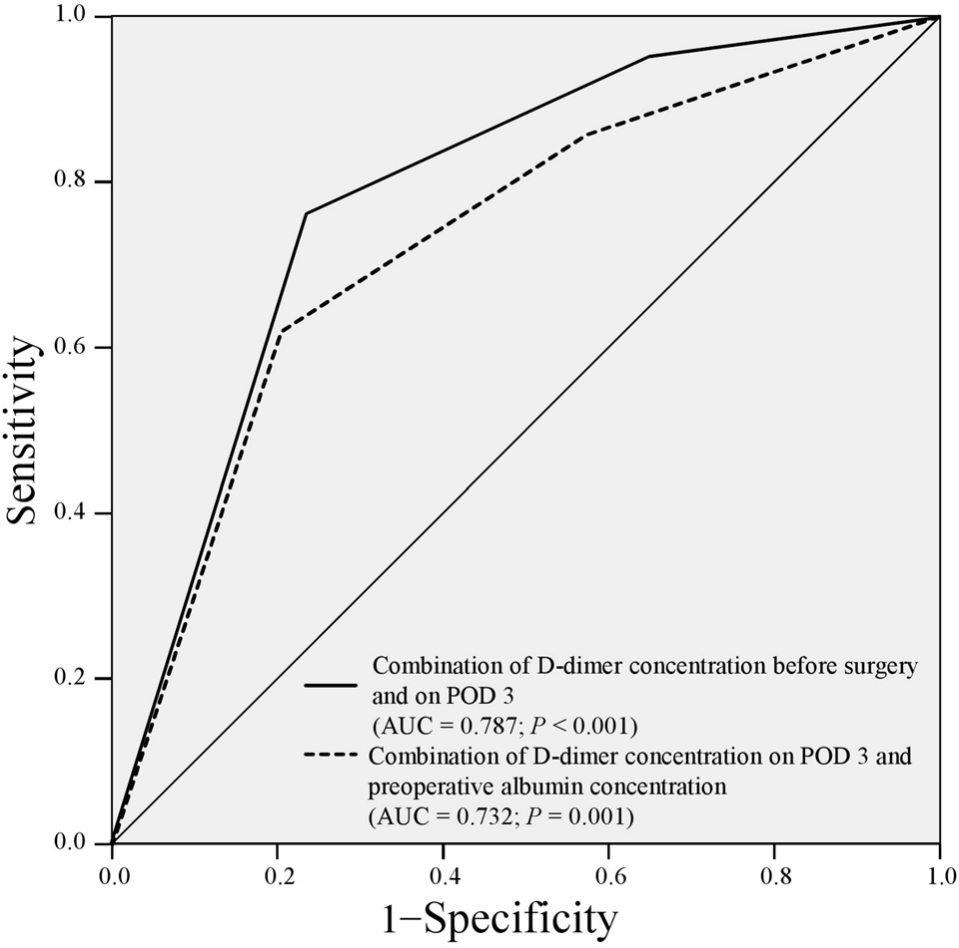
Areas under the curve of the combination of preoperative D-dimer or preoperative albumin concentration and D-dimer on POD 3 for the prediction of postoperative deep vein thrombosis in patients who underwent hepatobiliary-pancreatic surgery *AUC* area under the curve; *POD* postoperative day

Multivariate analysis of the predictive risk factors for DVT in early postoperative period revealed that the D-dimer concentration on POD 3 was an independent risk factor for postoperative DVT (OR, 4.149; 95% CI 1.104–15.593; *P* = 0.035), along with the preoperative eGFR (OR, 4.858; 95% CI 1.571–15.026; *P* = 0.006) in patients who underwent HBP surgery (Table 4). We also performed logistic regression analysis to investigate the preoperative factors for predicting high D-dimer concentrations on POD 3. The results of this analysis are shown in Table 5. Preoperative albumin and D-dimer concentrations were identified as independent predictive factors of an increase in the D-dimer concentration on POD 3. Based on this finding, we evaluated the AUC of the combination of the preoperative D-dimer concentration or the preoperative albumin concentration and the D-dimer concentration on POD 3. The AUCs of the combination of preoperative D-dimer concentration or preoperative albumin concentration and D-dimer concentration on POD 3 were 0.787 (*P* < 0.001) and 0.732 (*P* = 0.001), respectively. The AUC of the combination of the D-dimer concentration before surgery and on POD 3 was slightly superior to that of the D-dimer concentration on POD 3 alone (Fig. 3).

**Table 4:** Univariate and multivariate analyses of predictive risk factors for deep vein thrombosis in the early postoperative period after hepatobiliary-pancreatic surgery

Variables	Univariate analysis			Multivariate analysis		
	Odds ratio	95% CI	*P*	Odds ratio	95% CI	*P*
Age (≥ 75 vs. < 75 years)	1.605	0.635–4.56	0.317			
Sex (male vs. female)	0.760	0.302–1.914	0.560			
BMI (≥ 22.4 vs. < 22.4 kg/m^2^)	0.799	0.319–2.004	0.633			
Diagnosis (malignant vs. benign)	4.531	0.584–35.143	0.148			
ASA classification (3 or 4 vs. 1 or 2)	0.689	0.239–1.987	0.490			
Antithrombotic medication before surgery (present vs. absent)	2.227	0.663–7.486	0.195			
Preoperative platelet count (≥ 215 vs. < 215 × 10^3^/μL)	1.266	0.508–3.151	0.613			
Preoperative CRP (≥ 0.5 vs. < 0.5 mg/dL)	1.505	0.508–4.459	0.461			
Preoperative PT (< 97.7% vs. ≥ 97.8%)	1.732	0.680–4.410	0.249			
Preoperative eGFR (< 70 vs. ≥ 70 mL/min/1.73 m^2^)	4.903	1.709–14.067	0.003	4.858	1.571–15.026	0.006
Hepatectomy with extrahepatic bile duct resection, including hepatopancreatoduodenectomy (present vs. absent)	7.350	2.421–22.316	< 0.001	3.657	0.959–13.945	0.058
Operative time (≥ 468 vs. < 468 min)	1.870	0.734–4.762	0.189			
Intraoperative blood loss volume (≥ 438 vs. < 438 mL)	2.078	0.796–5.425	0.135			
D-dimer on POD 1 (≥ 5.35 vs. < 5.35 μg/mL)	3.979	1.559–10.155	0.004	1.639	0.551–4.870	0.374
D-dimer on POD 3 (≥ 6.25 vs. < 6.25 μg/mL)	6.462	2.244–18.609	0.001	4.149	1.104–15.593	0.035
D-dimer on POD 5 (≥ 12.10 vs. < 12.10 μg/mL)	3.328	1.313–8.433	0.011	0.999	0.290–3.437	0.999

*DVT* deep vein thrombosis, *CI* confidence interval *BMI* body mass index; *ASA* American Society of Anesthesiologists, *CRP* C-reactive protein, *PT* prothrombin time, *eGFR* estimated glomerular filtration rate, *POD* postoperative day

**Table 5:** Univariate and multivariate analyses of the preoperative factors predicting high D-dimer levels on postoperative day 3 after hepatobiliary-pancreatic surgery

Variables	Univariate analysis			Multivariate analysis		
	Odds ratio	95% CI	*P*	Odds ratio	95% CI	*P*
Age (≥ 75 vs. < 75 years)	1.300	0.687–2459	0.421			
Sex (male vs. female)	1.199	0.643–2.236	0.568			
BMI (≥ 22.4 vs. < 22.4 kg/m^2^)	0.869	0.474–1.593	0.649			
Diagnosis (malignant vs. benign)	3.706	1.344–10.215	0.011	1.984	0.659–5.966	0.223
ASA classification (3 or 4 vs. 1 or 2)	1.813	0.946–3.475	0.073			
Antithrombotic medication before surgery (present vs. absent)	1.067	0.398–2.859	0.897			
Preoperative serum albumin (< 4.0 vs. ≥ 4.0 g/dL)	3.424	1.814–6.461	< 0.001	2.276	1.146–4.523	0.019
Preoperative platelet count (≥ 215 vs. < 215 × 10^3^/μL)	0.555	0.300–1.027	0.066			
Preoperative CRP (≥ 0.5 vs. < 0.5 mg/dL)	2.469	1.134–5.372	0.023	1.627	0.704–3.759	0.255
Preoperative PT (< 97.7% vs. ≥ 97.8%)	1.465	0.798–2.691	0.218			
Preoperative eGFR (< 70 vs. ≥ 70 mL/min/1.73 m^2^)	1.237	0.673–2.273	0.494			
D-dimer before surgery (≥ 0.45. vs. < 0.45 μg/mL)	3.664	1.848–7.264	< 0.001	2.243	1.054–4.773	0.036

*POD* postoperative day, *CI* confidence interval, *BMI* body mass index, *ASA* American Society of Anesthesiologists, *CRP* C-reactive protein, *PT* prothrombin time, *eGFR* estimated glomerular filtration rate

## Discussion

HBP surgeries are associated with serious and potentially fatal complications because they are among the most highly invasive of all gastroenterological surgeries. DVT is often asymptomatic in the early period and it usually occurs postoperatively, during the first 12 (median) days after surgery [2]. Untreated asymptomatic DVT can result in PE, which may progress to a life-threatening condition. Therefore, predicting the development of DVT during the perioperative period is important for avoiding this situation after HBP surgery. Several studies have demonstrated that D-dimer assays have a high negative predictive value, and that D-dimer is a sensitive but nonspecific marker of DVT [3, 7]. In fact, a perioperative elevated D-dimer concentration is reportedly a reliable predictive risk factor for postoperative DVT [8–10]. However, few reports have addressed the usefulness and optimal timing of D-dimer assay for predicting DVT during the perioperative period following HBP surgery. Moreover, which HBP surgical procedures are most strongly associated with the development of postoperative DVT remain unclear. We conducted the present study to address these issues.

This study is the first to demonstrate that the D-dimer concentration on POD 3 is a simple and readily available factor for early prediction of the development of DVT in patients following HBP surgery. D-dimer is a fibrin-related marker that serves as an indicator of coagulation activation and fibrinolysis. A high D-dimer concentration is recognized as a reliable marker for a hypercoagulable state [11]. The elevation of D-dimer continues for several days because its half-life in plasma is longer (range, 3–48 h) than that of other fibrin-related markers [12]. Accordingly, we presumed that the absolute value of D-dimer may not appreciably predict the development of DVT in the early postoperative period. The current study showed that the D-dimer concentration was significantly higher in patients with than in those without postoperative DVT before surgery and on PODs 1, 3, and 5, and that the D-dimer concentration increased gradually from preoperatively to POD 5, when its highest value was reached. However, the optimal timing of D-dimer measurement for the prediction of DVT was identified as POD 3, which showed the greatest disparity in the D-dimer concentration between the patients with and those without DVT, and not POD 5, which showed the highest absolute value of the D-dimer concentration. This suggests that the disparity in the D-dimer concentration between the two groups is more important than the absolute value for predicting DVT during the early postoperative period. Yang et al. [13] reported that a highly significant deviation of the plasma D-dimer concentration between patients with and those without DVT was found on POD 3, with an AUC of 0.924 in the ROC analysis, in younger patients who underwent open reduction and internal fixation of lower limb fractures. Likewise, in another study, the greatest disparity of the D-dimer concentration between patients with and those without postoperative VTE after lung surgery was observed on POD 3, with significance, and the AUC on POD 3 was highest for the prediction of postoperative VTE [14]. Moreover, the concentration of fibrinogen degradation products, which are fibrin-related factors, was significantly higher in patients with than in those without VTE only on POD 3 after lung surgery [14]. These reports support the usefulness of D-dimer measurement on POD 3 for the prediction of DVT after HBP surgery, as found in the current study. Preoperative factors, particularly preoperative D-dimer concentration, are believed to affect the development of postoperative DVT. Similarly, in this study, the preoperative D-dimer concentration was identified as a factor predictive of an increase in the D-dimer concentration on POD 3. Therefore, we evaluated the AUC of the combination of D-dimer concentration before surgery and on POD 3 under the hypothesis that accurate prediction was possible by combining pre- and postoperative D-dimer concentrations. However, the results showed that the AUC of the combination of D-dimer concentration before surgery and on POD 3 was only slightly superior to that of the D-dimer concentration on POD 3 alone. Although the development of postoperative DVT is multifactorial, which might affect the AUC values, our results suggest that the measurement of D-dimer concentration on POD 3 alone is an option for detecting postoperative DVT.

Transient dysfunction of the liver, especially synthetic dysfunction, occurs after hepatectomy, which impairs postoperative coagulability. Patients who have undergone hepatectomy suffer a brief hypercoagulable state despite an elevation in the prothrombin time–international normalized ratio [15]. Thus, these patients are considered more predisposed to postoperative VTE than patients who have undergone other gastroenterological surgeries associated with less impairment of coagulability. Post-hepatectomy VTE is closely associated with the extent of liver resection. Studies have shown that patients who underwent liver resection with obesity or extended liver resection were at higher risk of VTE [16, 17]. Furthermore, with respect to the association between the operative approach and the incidence rate of VTE following hepatectomy, open major hepatectomy is the operative approach with the highest risk for postoperative VTE among all operative approaches to hepatectomy, including combinations of an open or minimally invasive approach and major or minor hepatectomy [18]. Extended hepatectomy with extrahepatic bile duct resection or hepatopancreatoduodenectomy is the most invasive, with both a longer operative duration and greater intraoperative blood loss than simple hepatectomy without extrahepatic bile duct resection. Furthermore, both a prolonged operation time and intraoperative blood transfusion are risk factors for VTE in HBP surgery [19–21]. Therefore, we assumed that hepatectomy (S4a + S5 resection or hemihepatectomy + caudate lobectomy) with extrahepatic bile duct resection, including hepatopancreatoduodenectomy, was a major risk factor for the development of DVT after HBP surgery. Our results showed that hepatectomy (S4a + S5 resection or hemihepatectomy + caudate lobectomy) with extrahepatic bile duct resection, including hepatopancreatoduodenectomy, was consistently the highest-risk procedure for the development of postoperative DVT among the many HBP surgical procedures. However, the present study could not confirm whether hepatectomy (S4a + S5 resection or hemihepatectomy + caudate lobectomy) with extrahepatic bile duct resection, including hepatopancreatoduodenectomy, was an independent risk factor for the development of DVT after HBP surgery, although it tended to show significance. The small sample size in the present study might have affected this result.

This study has two main limitations. First, it was a retrospective study involving a small cohort of only Asian individuals, which may have led to bias, prompting the need for a large-scale, prospective study to confirm our results. Second, we performed only ultrasonography to assess the development of postoperative DVT, based on a previous report on the diagnosis of DVT by Di Nisio [22]. However, using only ultrasonography might have affected the diagnostic accuracy of detecting postoperative DVT. Therefore, preoperative ultrasonography and examinations using other modalities should be considered in future studies for accurate evaluation of the development of postoperative DVT.

In conclusion, measuring the D-dimer concentration on POD 3 is a simple, convenient, cost-effective, and useful tool for the early prediction of DVT after HBP surgery. Moreover, high D-dimer concentrations on POD 3 could be predicted by albumin levels or D-dimer preoperatively. Patients with a high D-dimer concentration, especially those with both a high D-dimer concentration on POD 3 and extended hepatectomy with extrahepatic bile duct resection or hepatopancreatoduodenectomy, should receive early postoperative thromboprophylaxis to prevent postoperative DVT.

## Data Availability

All authors are available for the data of this study.
